# MatrixDB 2024: an increased coverage of extracellular matrix interactions, a new Network Explorer and a new web interface

**DOI:** 10.1093/nar/gkae1088

**Published:** 2024-11-18

**Authors:** Kasun W Samarasinghe, Max Kotlyar, Sylvain D Vallet, Catherine Hayes, Alexandra Naba, Igor Jurisica, Frédérique Lisacek, Sylvie Ricard-Blum

**Affiliations:** SIB, Swiss Institute of Bioinformatics, Geneva, Switzerland; Osteoarthritis Research Program, Division of Orthopedic Surgery, Schroeder Arthritis Institute and Data Science Discovery Centre for Chronic Diseases, Krembil Research Institute, University Health Network, Toronto, ON M5T 0S8, Canada; Institut de Biologie Structurale, UMR 5075, CEA, CNRS, Université Grenoble Alpes, Grenoble 38000, France; SIB, Swiss Institute of Bioinformatics, Geneva, Switzerland; Department of Physiology and Biophysics, University of Illinois Chicago, Chicago, IL 60612, USA; Osteoarthritis Research Program, Division of Orthopedic Surgery, Schroeder Arthritis Institute and Data Science Discovery Centre for Chronic Diseases, Krembil Research Institute, University Health Network, Toronto, ON M5T 0S8, Canada; Departments of Medical Biophysics and Computer Science, and the Faculty of Dentistry, University of Toronto, Toronto, Ontario, Canada; Institute of Neuroimmunology, Slovak Academy of Sciences, Bratislava, Slovakia; SIB, Swiss Institute of Bioinformatics, Geneva, Switzerland; Institut de Chimie et Biochimie Moléculaires et Supramoléculaires (ICBMS), UMR 5246, CNRS, Université Lyon 1, Villeurbanne 69622, France

## Abstract

MatrixDB, a member of the International Molecular Exchange consortium (IMEx), is a curated interaction database focused on interactions established by extracellular matrix (ECM) constituents including proteins, proteoglycans, glycosaminoglycans and ECM bioactive fragments. The architecture of MatrixDB was upgraded to ease interaction data export, allow versioning and programmatic access and ensure sustainability. The new version of the database includes more than twice the number of manually curated and experimentally-supported interactions. High-confidence predicted interactions were imported from the Integrated Interactions Database to increase the coverage of the ECM interactome. ECM and ECM-associated proteins of five species (human, murine, bovine, avian and zebrafish) were annotated with matrisome divisions and categories, which are used for computational analyses of ECM -omic datasets. Biological pathways from the Reactome Pathway Knowledgebase were also added to the biomolecule description. New transcriptomic and expanded proteomic datasets were imported in MatrixDB to generate cell- and tissue-specific ECM networks using the newly developed in-house Network Explorer integrated in the database. MatrixDB is freely available at https://matrixdb.univ-lyon1.fr.

## Graphical Abstract

**Graphical Abstract:**
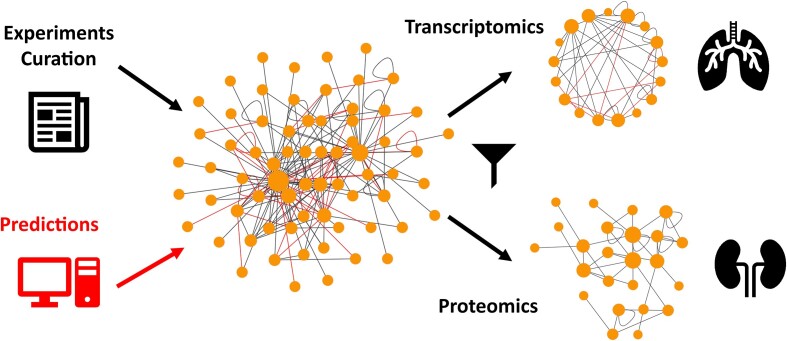

## Introduction

MatrixDB is a curated database focused on interactions mediated by the components of the extracellular matrix (ECM), namely proteins, proteoglycans, glycosaminoglycans, and bioactive ECM fragments collectively referred to as matrikines or matricryptins (1). One of the specific features of MatrixDB is the curation of ECM interactions at several molecular levels, namely matrikines/matricryptins (bioactive fragments, e.g. endostatin), individual polypeptide chains (e.g. collagen α chains), and native multimeric ECM molecules (e.g. trimeric collagens). The database is freely available through a web interface (https://matrixdb.univ-lyon1.fr), does not require any login or registration and is not password-protected. MatrixDB has been regularly updated since its creation in 2009 (2–5). MatrixDB is one of the services of the French node of ELIXIR (https://elixir-europe.org/about-us/who-we-are/nodes/france), and an active member of the International Molecular Exchange (IMEx) consortium (https://www.imexconsortium.org/) (6), which is a Global Core Biodata Resource (https://globalbiodata.org). Interaction data available in MatrixDB are manually curated from the literature via the curation interface of the IntAct database (7) following the IMEx curation rules (8).

The architecture of MatrixDB was designed to improve reusability (ease of data export and implementation of programmatic access) and long-term sustainability (allowing for versioning, and strongly limiting dependencies to unsupported technology), and to automate data import from an extended number of sources as detailed below. The content of the database was expanded, and MatrixDB contains twice as many experimentally-supported and manually curated interactions as the previous release (5). High-confidence predicted interactions from the Integrated Interactions Database (IID) (9), were added to increase the coverage of the ECM interaction networks generated with the new in-house ‘Network Explorer’ tool integrated in the database. Matrisome divisions and categories (10), now a standard in computational analyses of ECM -omic datasets (11,12), were added to annotate ECM and ECM-associated proteins of five species together with biological pathways imported from the Reactome Pathway Knowledgebase (13). Transcriptomic data and an expanded ECM proteomic dataset were imported from Bgee, an integrated curated expression atlas (14), and MatrisomeDB 2.0 (15) respectively to increase the coverage of cell- and tissue-specific interaction networks generated with the Network Explorer. Selected interaction data can now be exported in a tabular format and the corresponding networks in Cytoscape format in addition to image capture for further analyses.

## Architecture and management system of the database

The new system architecture comprises two main subsystems, data integration and the Web portal (see Supplementary Figure S1, Supplementary Methods, https://github.com/glyco-expasy/matrixdb-api and https://github.com/glyco-expasy/matrixdb-ui for details). This version (release 4.0) is and the future ones will be archived in the same GitHub repository. The database content was shifted from the unsupported ACEDB database manager software (16) to a NoSQL design (MongoDB, https://www.mongodb.com). A simple data model was defined to represent the entities of MatrixDB, which ideally could extend to other formats/models such as RDF (Resource Description Framework). It is implemented using MongoDB v.6.0, a NoSQL database.

## Data integration and visualization

The database was built by integrating data from several open biological data sources as detailed below. This integration process followed the Extract Transform Load (ETL) pattern, where data were extracted from external Application Programming Interfaces (APIs) or other forms of distributions (zip archives), then transformed according to the data model and loaded to the database. The data integration framework was developed in Python to perform the ETL process, considering the increased volume of data to be processed and the variety of input data formats. For each external data source, a data integration pipeline consisting of independent ETL subcomponents was developed, with parallel processing, whenever necessary to handle large data volumes. All pipelines were designed to maximize reusability in the data integration process, and ease regular updates of MatrixDB content. Third-party JavaScript libraries, Cytoscape (17), Mol* (18) and D3 (https://d3js.org/) were used to implement visual components (Supplementary Table S1).

## An expanded interaction dataset

### Experimentally-supported interaction data

**Figure 1.**
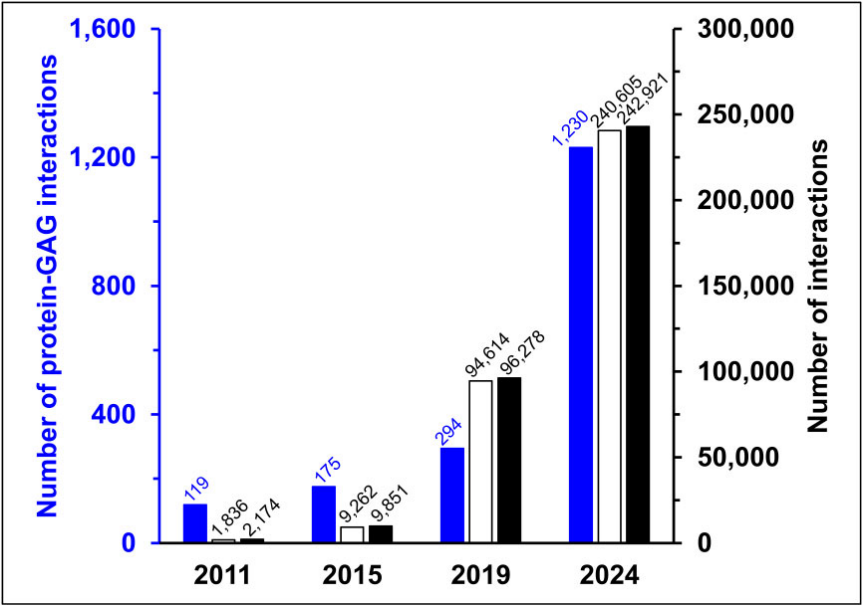
Number of experimentally-supported interactions in MatrixDB releases. The plot shows the increase in the total number of experimental interactions (black), and in the number of experimental protein-protein (white) and protein-GAG (blue) interactions. The ‘protein’ category includes monomeric proteins and individual polypeptide chains of multimers (e.g. α chains of collagens).

The new interaction dataset (release 4.0) comprises experimentally-supported interactions manually curated from publications by MatrixDB, and interactions involving at least one ECM component imported from IntAct (release 2024–02) (7), and other databases of the IMEx consortium (6). Orthologous experimental protein-protein interactions are not inferred to human species in the new version of MatrixDB. The new experimental interaction dataset comprises ∼243 000 interactions, a two-fold increase in the number of manually curated and experimentally-supported interaction data compared to the previous version (5) (available at http://v1.matrixdb.univ-lyon1.fr/) (Figure 1). This increase is mainly due to the import from IntAct database of large protein-protein interaction datasets including tens of thousands of interactions, some of them involving ECM proteins, such as BioPlex 2.0 (19), resulting in a 2.5-fold increase in the number of protein-protein interaction data. However, there is a ∼4-fold increase in the number of GAG-protein interactions, from 294 to 1230, in release 4.0 (Figure 1) with a 3-fold increase in the number of heparin interactions (from 251 to 766). There is also a significant increase in the number of interactions of ECM proteins (e.g. a 4.4-fold increase in the number of interactions of lysyl oxidase-like 2, from 12 to 53 in the 4.0 release). MatrixDB curated dataset and the entire interaction dataset available in MatrixDB can be downloaded from the ‘Download’ tab under the MITAB 2.7 format.

### Predicted protein–protein interactions

High-confidence computationally predicted protein–protein interactions (151 132) from the Integrated Interaction Database (9), a member of the IMEx consortium, were added to MatrixDB. These high-confidence interactions (see Supplementary Methods for details) were predicted by state-of-the-art computational methods (20–23). They are clearly labeled as such in the database, and can be included, or not, in the networks generated with the Network Explorer.

### Display of interaction data

Interaction data are displayed in the ‘Biomolecule’ page as an interactive table in a first tab (Interaction list view), and an interactive network in a second tab (Network view). The number of experimentally supported and predicted interactions of the biomolecule is indicated at the top. The table comprises six columns including the names and identifiers of the participants, the nature of the interaction (direct or indirect), the molecular interaction (MI) score imported from the IntAct database (7) for experimental interactions, and the type of interaction (experimental interactions in black or predicted interactions in red). Clicking on one interaction opens an ‘Association’ page that provides the identifiers of the participants, the MI score and the list of experiments supporting the interaction. Clicking on one experiment opens an ‘Experiment’ page where the interaction detection method, the participant detection method, the binding region, the stoichiometry, kinetics (association and dissociation rates), the equilibrium constant (affinity), and the effect of mutations on the interaction are displayed when available.

## Annotation of biomolecules

Biological pathways were imported from the Reactome Pathway Knowledgebase (13), and displayed at the bottom of the ‘Biomolecule’ page together with Gene Ontology (GO) terms (24,25) and UniProtKB keywords (26). ECM and ECM-associated proteins were first annotated with matrisome divisions (core matrisome and matrisome-associated), and then with one of the six matrisome categories (ECM glycoproteins, collagens and proteoglycans for core matrisome components, and ECM-affiliated, ECM regulators, and secreted factors for matrisome-associated components), which are now broadly adopted for -omic analyses of ECM datasets. The following lists were used for matrisome annotations: human and murine matrisomes (27), bovine matrisome (28), avian matrisome (29) and zebrafish matrisome (30). Matrisome annotations are displayed on the upper right corner of the ‘Biomolecule’ page. An additional, manual, annotation called ‘MatrixDB ECM’ includes proteins, which are not annotated as matrisome proteins, but are annotated with at least one of selected UniProtKB keywords (Extracellular space/secreted: KW-0964, Extracellular matrix: KW-0272, and Basement membrane: KW-0084) or GO term Cellular Component (Extracellular space: GO:0005615, Extracellular region: GO:0005576, Extracellular matrix: GO:0031012, and Basement membrane: GO:0005604). The list of ‘MatrixDB ECM’ proteins is available in the ‘Download’ tab.

## Basic search and advanced search

Indexing and searches are performed with a configured version of Solr v.9.5 (https://solr.apache.org/), an open-source community software. Basic search queries all fields of the database, and includes free text, biomolecule and gene names, accession numbers of UniProtKB (26), Complex Portal (31) and Chemical Entities of Biological Interest (ChEBI) (32) and PMID identifiers. By default, the ten most relevant results (award icon, Supplementary Figure S2) are displayed where relevance is first defined by the field of the database where the searched term is found. For example, the term found in a biomolecule name is considered more relevant than in an annotation (see Supplementary Methods for further details). The eye icon allows users to display all the results (ten items per page), whereas the filter icon allows them to select a biomolecule category and/or a species (Supplementary Figure S2). Advanced search allows users to retrieve biomolecules using their names, identifiers, UniProtKB keywords, GO terms, matrisome divisions and categories, and Reactome biological pathways. The database can also be queried using a combination of species AND GO term or species AND matrisome division/category. The Biomolecule search displays biomolecules included in the database whether they have binding partners or not.

## Transcriptomic and proteomic data

Transcriptomic data were imported from the Bgee expression atlas (14), comprising curated and re-annotated GTEx (Genotype Tissue Expression) samples based on healthy expression data (14) to replace GTEx data formerly integrated in MatrixDB. An expanded and curated ECM proteomic dataset including over 150 000 protein entries from 42 studies on the ECM on over 25 different healthy and diseased tissues and organs was imported from MatrisomeDB 2.0 (15). These experimental datasets increase the coverage of cells and tissues used to filter the networks generated with the Network Explorer (see below). Transcriptomic data (transcripts per million) are displayed in anatomograms imported from Gene Expression Atlas (https://www.ebi.ac.uk/gxa) in a first tab and proteomic data (normalized spectral abundance factor, NSAF) as heatmaps in a second tab.

## Network Explorer

User-selected biomolecules can be imported in the Network Explorer interface from the ‘Biomolecule’ page by clicking on the ‘+’ button next to the green Network Explorer icon in the header. Once the biomolecule is added to the Explorer, the icon turns from green to white and clicking on it gives access to the Explorer. Biomolecules can also be imported from the ‘Network Explorer’ page, and the top window can be used to search the database. In contrast to the previous navigator of MatrixDB, the Network Explorer includes by default interactions between partners. They can be excluded by ticking the ‘Exclude partner interactions’ box. The Network Explorer remembers the selection of biomolecules in the browser session. Filters are available as a right menu to select network participants (e.g. those annotated as ECM biomolecule, in either MatrixDB ECM or matrisome lists), and interactions based on experimental and/or predicted interactions, expression level (transcriptomic data) or protein abundance (proteomic data). An additional filter allows user to select the interaction detection method. Filters can be reversed to display the unfiltered network. In addition to an option for image capture (camera icon), selected interaction data can be exported in tabular format (table icon) and the corresponding networks in Cytoscape format (Cytoscape icon) for further analyses. The search in the Network Explorer is restricted to biomolecules which are involved in at least one interaction.

### Interaction networks including various proteoforms of ECM proteins

**Figure 2.**
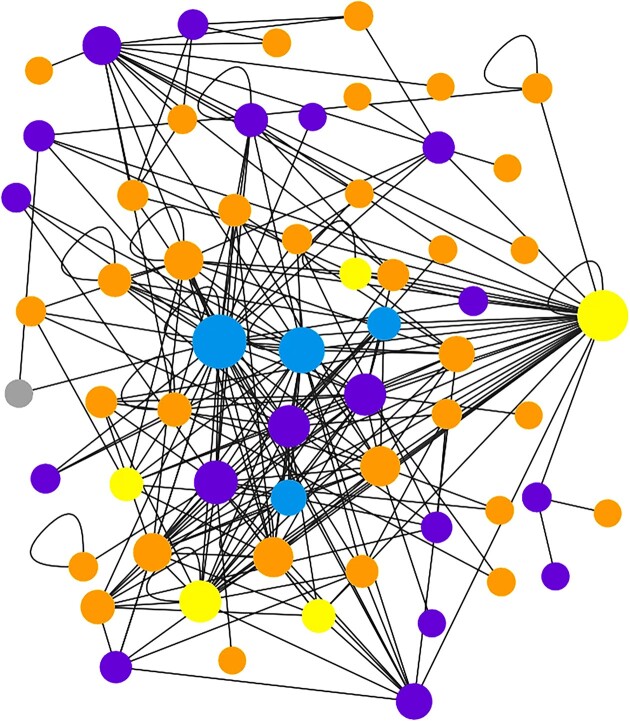
Interaction networks of several molecular proteoforms of human collagens. Network of selected native trimeric collagens, individual collagen a chains and collagen matrikines/matricryptins. It has been built using the Network Explorer using ‘Collagen’, ‘Multimers’ and ‘*Homo sapiens*’ as filters. The biomolecules are color-coded (protein: orange, glycosaminoglycan (GAG): blue, multimer: purple, protein fragment (PFRAG): yellow, grey: small molecule).

One of the specific features of MatrixDB is to ease the retrieval of interactions curated at several molecular levels and to visualize them as interaction networks reflecting what happens *in vivo*. This is illustrated in Figure 2, which recapitulates interactions established by several native trimeric collagen molecules (purple), which are rarely included in interactomes, individual collagen α chains (orange), collagen bioactive fragments (matrikines/matricryptins, yellow) and glycosaminoglycans (blue).

### Increased coverage of ECM protein networks and tissue-specific networks

**Figure 3.**
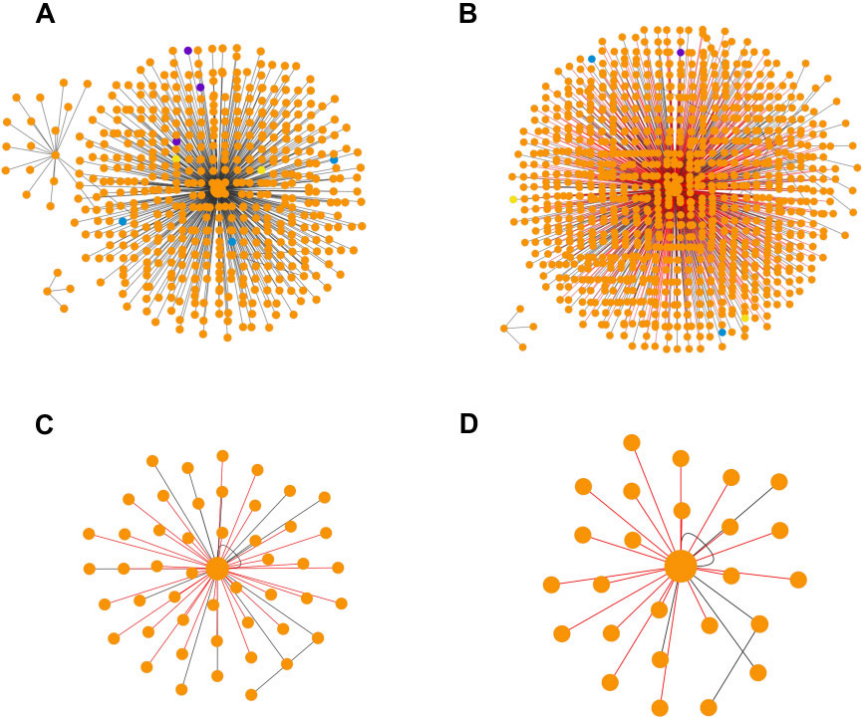
Interaction networks of human fibronectin generated using the Network Explorer of MatrixDB. (**A**) Fibronectin network comprising experimental interactions only. The small network located in the bottom left corner comprises an isoform of fibronectin (P02751–12) and its three partners. The biomolecules are color-coded (protein: orange, glycosaminoglycan (GAG): blue, multimer: purple, protein fragment (PFRAG): yellow). (**B**) Fibronectin network comprising both experimental (black links) and predicted interactions (red links). (**C**) Fibronectin network in blood vessel generated by filtering the entire network with proteomic data (normalized spectral abundance factor 501). (**D**) Fibronectin network in the eye generated by filtering the entire network with proteomic data (normalized spectral abundance factor 501).

The interaction network of human fibronectin generated with the Network Explorer highlights its increased coverage due to the addition of predicted interactions. There is a 2-fold increase in the size of the fibronectin network upon addition of predicted interactions (638) to experimental ones (512) (Figure 3A and B). Filtering the entire fibronectin network using proteomic data at the tissue and NSAF levels results in fibronectin networks of various size depending on the tissue (e.g. blood vessel and eye, Figure 3C and D, respectively).

## Conclusion

The architecture and the management system of MatrixDB database were entirely rebuilt to replace the previous version, which was no longer supported, and ease data import and export as well as updates of MatrixDB content. Furthermore, the system architecture is designed with scalability as a core consideration, addressing both data integration and the web portal application in which a high volume of concurrent requests can be handled via the API framework, thereby accommodating an expanding database. The new version of MatrixDB includes a 2-fold expanded experimental dataset as well as predicted protein-protein interactions, curated transcriptomic data, an expanded ECM proteomic dataset, a new Network Explorer, and several export modes of user-selected interactions for further computational analysis.

On-going work aims to complete the curation of the glycosaminoglycan interaction dataset, MatrixDB being the primary database to specifically curate and report those interactions. We will also pursue the curation of low-throughput, biophysical, interactions of native ECM molecules and matrikines/matricryptins to build a comprehensive ECM interaction network including them. Mutations affecting interactions and associated with rare ECM genetic disorders (e.g. collagenopathies such as Ehlers-Danlos syndrome, osteogenesis imperfecta or epidermolysis bullosa) (33), will be mapped on tissue- and cell-specific ECM networks to determine how these networks are rewired by these mutations, and to decipher the molecular mechanisms altering ECM organization and function in these diseases.

## Supplementary Material

gkae1088_Supplemental_File

## Data Availability

MatrixDB is freely available at https://matrixdb.univ-lyon1.fr. It is distributed under the terms of the Creative Commons Attribution Licence CC BY 4.0.
