# Supplementary material for: MatrixDB 2024: an increased coverage of extracellular matrix interactions, a new Network Explorer and a new web interface
Source: Nucleic Acids Res. 2024 Nov 18;53(D1):D1677–82. doi: 10.1093/nar/gkae1088 (PMC11701626; doi:10.1093/nar/gkae1088)
Supplement: gkae1088_Supplemental_File [file gkae1088_supplemental_file.pdf]

SUPPLEMENTARY MATERIAL

MatrixDB 2024: an increased coverage of extracellular matrix interactions, a new Network Explorer and a new web interface

Kasun Wijesiriwardana Samarasinghe[1], Max Kotlyar[2], Sylvain D. Vallet[3], Catherine Hayes[1], Alexandra Naba[4], Igor Jurisica[2], Frédérique Lisacek[1*], and Sylvie Ricard-Blum[5*]

SUPPLEMENTARY METHODS

Web portal

The Web portal application is mainly composed of a backend system and a single page frontend web application. The backend system interacts with the database and exposes the data in a meaningful manner such that the web interface can organize and visualize data. It consists of several sub-components as illustrated in Supplementary Figure S1. It exposes a REST API, as the standard interface for the frontend web application or to any other application, which intends to access MatrixDB data. It is implemented in Python v.3.11, along with Flask 3.0 API (https://flask.palletsprojects.com/en/latest/api/) as the API layer implementation.
The Web portal application is developed as a Single Page Application (SPA), which interacts with the MatrixDB API and retrieves the required data to produce the relevant pages. It is implemented with the React framework v.18.2 (https://react.dev/), in typescript. It uses the material UI, a standard reusable web component library to implement the pages and visual arrangement. The modular structure of the API, combined with a caching layer, optimizes resource usage and enables horizontal scaling, ensuring the system can efficiently support increasing demand over time.

High-confidence predicted interactions (PPIs)

Prediction studies defined their high confidence PPIs as follows:
- Rhodes *et al.* (*Nat. Biotechnol. 2005 23: 951-959*) used a likelihood ratio cut-off of 381
- Lefebvre *et al.* (*In: Systems Biology and Computational Proteomics. Springer Berlin Heidelberg, Berlin, Heidelberg, 2007, pp. 42–56*) used a probability greater than 0.5
- Elefsinioti *et al.* (*Mol. Cell. Proteomics 2011 10 : M111.010629*) used probabilities greater than 0.7
- Zhang *et al.* (Nature 2012 490 : 556-560) used likelihood ratio of at least 600
- Kotlyar *et al.* (*Nat. Methods 2015 12: 79-84*) used false discovery rate < 0.6
Most studies, including us, interpreted "confidence" by calculating a Bayesian probability of interaction: P(interaction | evidence) ~= likelihood * prior ~= P(evidence | interaction) * P(interaction) . And P(interaction) = |estimated size of human interactome| / |number of human protein pairs|. For example, Zhang *et al.* assumed

that there are ~650K human PPIs, resulting in a prior of about 1/600, and then their high confidence threshold of likelihood=600 corresponded to P(interaction|evidence) = 0.5. While this may seem low, it is an improvement in probability from 1/600 to 1/2. Also, 650K PPIs is an under-estimation, so the real P(interaction|evidence) may be much higher than 0.5.

Ranking search results by relevance

Search results are collected and ranked using the indexing system of Solr so that free text search is performed based on a weighed scoring mechanism that can be defined in this software. Those pre-defined weights reflect biomolecule attributes resulting in a search score used to rank the results. Solr searching capabilities span fielded search, Boolean queries, phrase queries, fuzzy queries, spell check, wildcards, grouping, auto-complete and others across different types of data. The index of a search term is therefore linked with other indices and a hierarchy was defined to keep biomolecule names at the top and associated annotations below.

SUPPLEMENTARY FIGURES

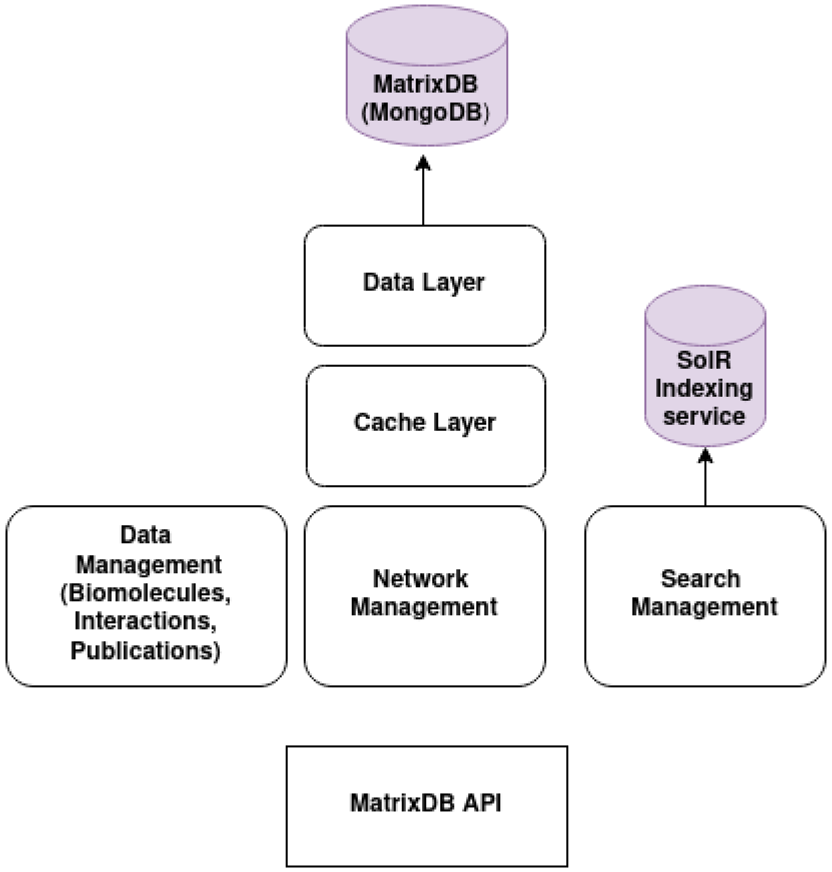

Supplementary Figure S1: **System architecture**. There are three main management components (data, network and search), which handle different aspects of the Web portal.

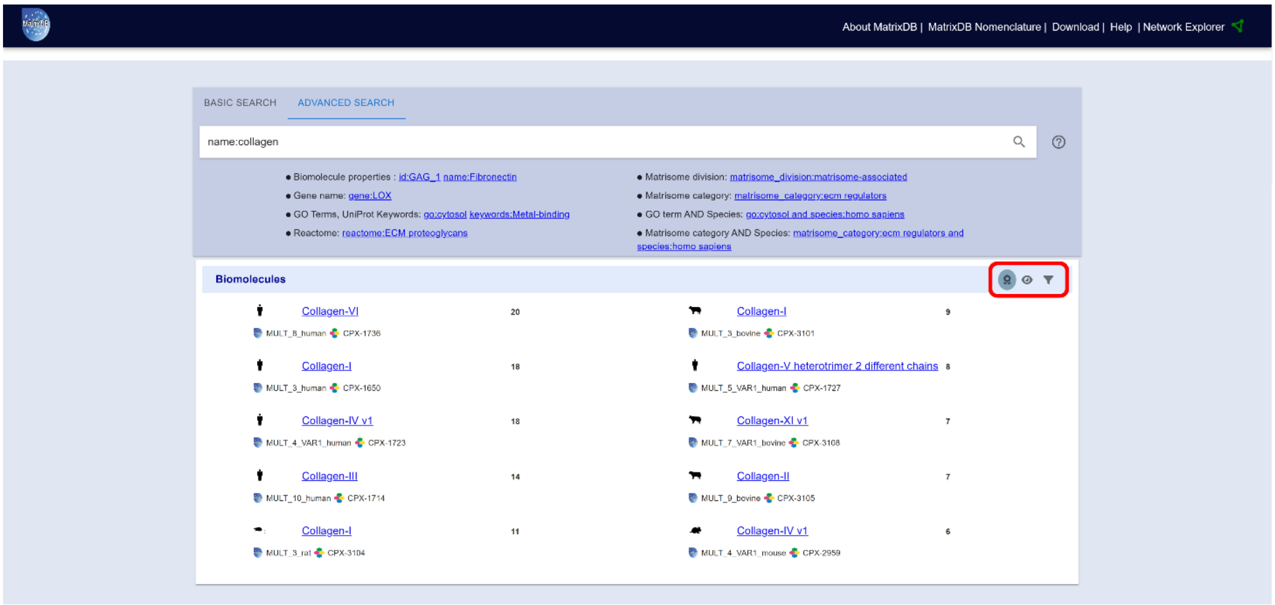

Supplementary Figure S2: Querying MatrixDB database using basic and advanced search. The icons circled in red allow users to access the most relevant results (award icon), to display all the result pages (eye icon), and to select a biomolecule category and/or a species (filter icon).

SUPPLEMENTARY TABLES

Name of libraries	Use in MatrixDB	Reference
**Cytoscape.js**, a graph theory library for visualization and analysis	Network visualization with most of the standard Cytoscape functionalities and compatible formats for export and reuse	*Franz et al., 2016 Bioinformatics 32: 309-11*
**Mol***, a 3D structure viewer	Visualization of 3D structures from the Protein Data Bank in Europe in Biomolecule pages to replace outdated LiteMol (*Sehnal et al., 2017, Nat. Methods 14:1121-1122*)	*Sehnal et al., 2021 Nucleic Acids Res. 49(W1):W431-W437*
**D3** (or D3.js), JavaScript library for visualizing data	Graphs and charts for statistics display and other visualization-related components	https://d3js.org/

Supplementary Table S1: Third-party JavaScript libraries used to implement visual components in MatrixDB.
